# Acute mountain sickness predicts the emotional state of amateur mountaineers

**DOI:** 10.1038/s41598-024-55291-3

**Published:** 2024-02-27

**Authors:** Zhengyang Zeng, Yun Zhou, Lun Li

**Affiliations:** School of Physical Education, China University of Geosciences (Wuhan), Wuhan, 430074 Hubei China

**Keywords:** Psychology, Psychiatric disorders

## Abstract

Research on amateur mountaineers is scarce, and this study aims to delve into the emotional experiences of ten amateur mountaineers during their ascent using the "Befindlichkeitsskala" (BFS) and Lake Louise Acute Mountain Sickness scoring system (LLS). These subjects were exposed to altitudes of 3140 m, 4300 m, and 5276 m, respectively. We found that LLS scores were negatively correlated with positive emotions (*β* = −27.54, *p* < 0.05) and positively correlated with negative emotions (*β* = 21.97, *p* < 0.05). At an altitude of 4300 m, individuals with AMS exhibited significant differences in depression, anger, excitement, and inactivity compared to climbers without AMS. Upon returning to 3140 m after completing the climb, significant differences were observed in emotions such as happiness, calmness, anger, excitement, and depression. Throughout the three-day climb, noteworthy differences emerged in activity, happiness, calmness, inactivity, positive emotions (*p* < 0.01), negative emotions, and overall emotional scores (*p* < 0.05). Our study suggests a decline in the emotional well-being of amateur climbers with increasing altitude, highlighting AMS as a pivotal predictive factor for emotional experiences while climbing.

## Introduction

Currently, there has been a notable surge in the number of people engaging in activities such as mountaineering and hiking in high-altitude areas. A significant proportion of these individuals ventured into highlands for the first time and lacked prior experience in sports activities at elevated altitudes^1^. Moreover, high-altitude areas present a pressurized environment, and at altitudes exceeding 2500 m, various physiological and psychological changes can occur^2^. Given the combined influence of multiple factors, engaging in outdoor activities for the first time in high-altitude areas poses numerous risks^3^.

In high-altitude areas, the impact of the natural environment on the human body is multifaceted, encompassing factors such as low atmospheric pressure, low partial pressure of oxygen and cold. Notably, a reduction in atmospheric pressure leads to a conspicuous low-oxygen environment, with physiological, biochemical, and adaptive changes in the human body becoming increasingly apparent at altitudes exceeding 2500 m. Engaging in sports in plateau regions characterized by low pressure and low oxygen introduces two distinct challenges compared to plains: the low-oxygen load associated with exercise and the low-oxygen load specific to high-altitude environments. These challenges prompt physiological habituation and adaptation within various bodily systems, including respiration and circulation^4^. During this period, the body is regulated by the neuroendocrine system through a series of compensatory adaptive changes, which include a hypoxic ventilatory response, diaphoretic response and physiological and biochemical changes. These encompass hypoxic ventilatory responses, diuresis, increased cardiac output, improved oxygen-bearing capacity and cerebral blood flow, and stabilization of hypoxia-inducible factor 1α (HIF-1α)^5^. However, individuals who ascend rapidly to altitudes of 2500 m or above without acclimatization are at risk of developing AMS^6^, which can progress to life-threatening conditions such as high-altitude cerebral edema and high-altitude pulmonary edema. The most common symptom of AMS is headache, which is accompanied by gastrointestinal symptoms (nausea/vomiting/poor appetite), fatigue, and dizziness^7^. Factors influencing AMS include rapid ascent, dehydration, alcohol consumption, respiratory infections, and individual susceptibility^8^. Exposure to high altitudes, oxygen content, and environmental factors in high-altitude areas can also lead to anxiety, depression, anger, fatigue, and other emotional issues^9–11^, which can contribute to accidents and fatalities^12^.

Currently, studies on the emotional changes and factors affecting amateur mountaineers during mountaineering are rare. However, similar studies have shown that there is a deterioration in mood after exposure to high altitude, mainly in the form of anxiety and depression^10,13^. Increased altitude leads to AMS, which in turn affects the mood of climbers. Compared with patients in the no-AMS group, AMS patients in the AMS group tended to exhibit a more severe negative mood within 24–48 h of entering high altitude, with greater anger and depression and a lack of vitality. Additionally, compared with those in the no-AMS group, participants in the AMS group experienced increased restlessness and decreased vitality when the altitude rose from 3000 to 4050 m^19^. The main manifestations of the increase in the AMS group as the altitude increases are an increase in negative emotions and a decrease in positive emotions^14^; when individuals return to lower altitudes, some of the negative emotions persist for up to a year or more^15^. While there have been reports indicating an increase in negative emotions with increasing altitude, there is limited information on positive emotions. Moreover, the critical threshold of altitude for emotional changes has not been precisely defined. Reports suggest that emotional disorders occur at altitudes above 3000 m^16^. Shukitt-Hale, Banderet and Lieberman^17^ reported hostility, depression, and anxiety at altitudes above 3500 m, with a worsening trend as altitude increases. Shukitt and Banderet^18^, and Nelson^19^ reported that emotional disorders occurred above 4000 m.

While our understanding of HA medicine and emotional changes has improved over the past few decades, the majority of related research has been conducted using hypobaric chambers, with limited studies focusing on the application of real-world environments^11^. Moreover, existing research in this domain has predominantly centered on professional mountaineers, leaving a notable gap in studies addressing a broader and more diverse population of amateur outdoor enthusiasts. A lesser understanding of the emotional changes in the amateur mountaineer community during mountaineering will expose amateur mountaineers to more risks. Therefore, this study aimed to expand the group of amateur mountaineers and explore the emotional experiences and influencing factors of amateur mountaineers during mountaineering to reduce the risks faced by amateur mountaineers.

## Methods

### Participants

The study included ten amateur mountaineers with an average age of 22.7 ± 1.34 years, weight of 65.7 ± 10 kg, and height of 173.4 ± 7.96 cm. None of the participants had visited high-altitude areas in the past 3 months, and none had any history of smoking, alcohol consumption, or mountaineering above 5000 m. All participants were first-time high-altitude mountaineers. To ensure that participants have the basic physical fitness required for this climb and to guarantee the execution of the ascent with a focus on personal safety, participants are required to participate in a four-week physical fitness training program prior to climbing. The single training session consisted of 10 km of running/trail running and core strength training, twice a week for 1–2 h each time. To acclimatize to the mountain environment, a 10-km weight-bearing hike was conducted one week prior to departure.

### Study design

**Figure 1:**
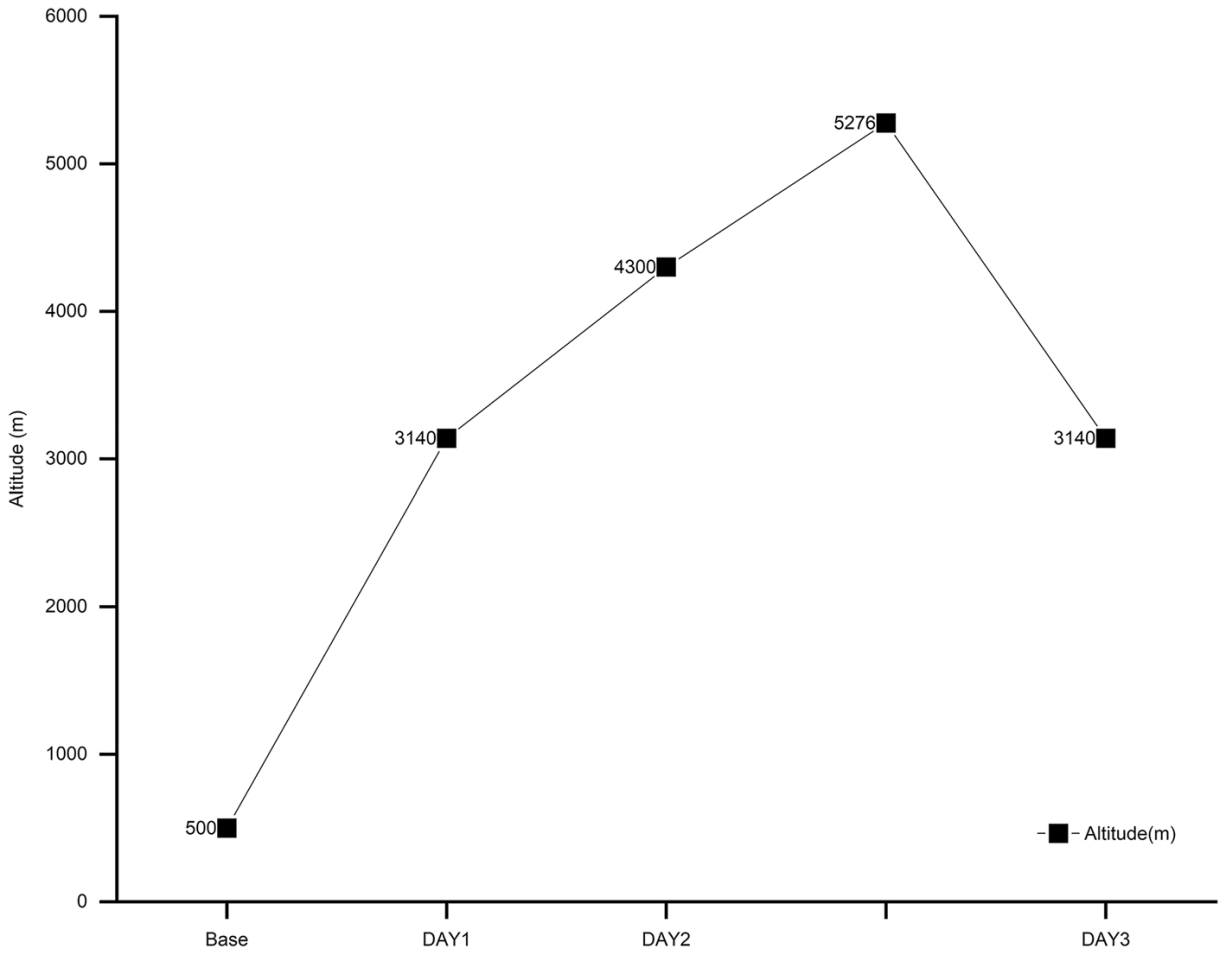
Elevation change.

This study was conducted in January 2021 in Sichuan Province, China, specifically at Mt. Siguniang Erfeng, which has an elevation of 5276 m. The participants were all from Wuhan, China, at an elevation of 23.3 m. To familiarize the participants with the research procedures, two simulated testing sessions were conducted one week before the actual mountain expedition, guided by designated personnel. The mountaineering expedition lasted for three days with the following itinerary: first day (Day 1): Departure from Chengdu to Siguniangshan town (elevation 3140 m) at 10:00, arriving at 15:00. Second day (Day 2): Departure from Siguniangshan town at 9:00, hiking to the Siguniangshan two-peak base camp (elevation 4,300 m), covering a total of 16 km. The arrival time was 16:45, with an average speed of 2.3 km/h. Third day (Day 3): The ascent started at 3:00, reached a summit at 7:15 (elevation 5276 m), and returned to the base camp at approximately 11:00, covering approximately 10 km with an average speed of 1.25 km/h. After a brief rest at the base camp, participants departed at 12:00 to return to the town of Siguniangshan, covering 16 km and arriving at 16:30, with an average speed of 3.55 km/h (Fig. 1). Participants, during the climb, carried only water, food, and personal belongings needed for the day, with backpack weights ranging from 2.5 to 5 kg. The BFS and the LLS were tested in a quiet and undisturbed environment 30 min after meals. The BFS was measured once a day, while the LLS was measured three times a day. The altitudes at which the BFS was tested were as follows: Day 1 (3140 m), Day 2 (4300 m), and Day 3 (3140 m). The altitudes at which the LLS was measured were as follows: Day 1 (3140 m), Day 2 (3140 m, 3750 m, 4300 m), and Day 3 (4300 m, 5276 m, 3140 m). The testing times on Day 3 were 3:30 AM, 8:00 AM, and 7:30 PM. To mitigate the impact of dietary discomfort, participants adhered to the principles of a high-sugar, low-fat, and multivitamin-supplemented diet during the hiking period. The participants avoided spicy foods and prepared their own meals on the road according to personal preferences.

### Acute mountain sickness

The Lake Louise Acute Mountain Sickness Scoring System (LLS) was used for screening AMSs^7,20^. The diagnostic criteria for AMS include a rapid ascent to high altitude within a short period accompanied by headache and at least one of the following symptoms: gastrointestinal symptoms, fatigue, or dizziness. The severity of symptoms was graded from 0 to 3, with 0 indicating no symptoms and 3 indicating severe symptoms. Self-reported scores in the Lake Louise Scoring System were categorized as mild (3–5 points), moderate (6–9 points), or severe (10–12 points). An LLS of 3 or higher is clinically diagnosed as AMS, while an LLS less than 3 indicates the absence of AMS.

### Emotions

The Befindlichkeitsskala Scale (BFS) was used for measuring emotions^21,22^. Compared to the Profile of Mood States (POMS), the BFS captures both positive and negative emotions, providing a more comprehensive assessment of emotional changes during mountaineering. The Chinese version of the BFS was revised by Gangyan and Zhijian^23^ and consists of eight subscales, each containing five items, for a total of 40 items. The scale is divided into two dimensions: an evaluative dimension (good/pleasant and bad/unpleasant) and an activation dimension (positive/negative). Cai^24^ retested the scale for reliability and validity. Two factors were extracted for factor analysis, with a cumulative contribution of 45.43%. Correlation tests between individual items and subscales showed that the correlation coefficients between the items and their respective subscales were greater than 0.5, except for the topics "distracted" in the agitation subscale and "angry" in the anger subscale, for which Cronbach's α = 0.67 and for which Cronbach's α = 0.5 in the anger subscale. Cronbach's alpha = 0.67 for the anger subscale, 0.69 for the agitation subscale, and 0.70 for all the other subscales, indicating that the Chinese version of the BFS showed good construct validity and internal consistency in the statistical analyses of scale correlations, item, scale correlations, and Cronbach's alpha coefficients. internal consistency. This study will focus on the activation dimension. The positive emotions subscale (BFS-P) included activity (energetic), happiness (carefree), thoughtfulness (reflective), and calmness (relaxed). The negative emotions (BFS-N) included anger (resentful), agitation (restless), depression (oppressed), and inactivity (inactive). The scale uses a Likert five-point scale (1 = not at all, 5 = completely).

### Statistical analysis

Due to the limited sample size, all analyses were conducted using nonparametric tests, and median values (P25, P75) were used for representation. The relationship between the LLS and BFS score was examined using Spearman's rank correlation test, and linear regression analysis was conducted to explore the factors influencing emotions. By determining whether the LLS was greater than or equal to 3, mountaineers were classified as AMS (AMS+) or de AMS (AMS−). The Wilcoxon rank-sum test was used to assess differences in BFS subscale scores between these two groups at different altitudes. The Friedman test was utilized to examine differences in BFS subscale scores across different altitudes. Pairwise comparisons were performed using the Nemenyi test based on the results of the Friedman test. IBM SPSS Statistics 26.0 (IBM, Chicago, IL, USA) was used for data processing, while OriginPro 2021 (OriginLab, Northampton, MA, USA) was used for data visualization and graphing.

### Ethics declarations

This study was conducted in accordance with the principles outlined in the Helsinki Declaration. It received approval from the Academic Integrity and Research Ethics Committee of China University of Geosciences (Wuhan). All participants were provided with and required to read and sign an informed consent form, ensuring their understanding of the study's purpose, procedures, potential risks, and their rights as participants.

## Results

The impact of acute mountain sickness on emotions: the results of Spearman's rank correlation analysis are presented in Table 1. The LLS was significantly negatively correlated with the BFS-P score (*r* = −0.43, *p* < 0.05), indicating a significant negative correlation between the LLS score and positive emotions. On the other hand, the LLS score was strongly significantly positively correlated with the BFS-N score (*r* = 0.74, *p* < 0.01), indicating that the LLS score was strongly positively correlated with negative emotions (Table 1).

**Table 1:** Correlation analysis between the LLS score and BFS.

	Median (P25, P75)	LLS	BFS	BFS-P	BFS-N
LLS	1 (0, 5.25)	1			
BFS	92.5 (87, 105.25)	0.31	1		
BFS-P	61 (46.5, 70.5)	−0.43*	0.43*	1	
BFS-N	34.5 (24, 44)	0.74**	0.44*	−0.53**	1

*p < 0.05.

**p < 0.01.

To further investigate the effects of AMS and altitude on emotions, a regression analysis was conducted. First, the Shapiro‒Wilk test was performed on the BFS-P and BFS-N to assess the normality of the distributions. The significance value for the BFS-P was 0.069, indicating that it follows a normal distribution, as it was greater than 0.05. For BFS-N, the significance value was 0.004, with a kurtosis of -0.305 and skewness of 0.854. While the data were not perfectly normal, the absolute values of kurtosis and skewness, both less than 10 and 3, respectively, are generally acceptable for a normal distribution^25^. Using AMS, LLS score, and altitude as independent variables and BFS-P and BFS-N as dependent variables, linear regression analysis was performed, as presented in Table 2. The F values for BFS-P and BFS-N were 5.31 and 16.41, respectively, with p values less than 0.01. The adjusted R-squared values were 0.31 and 0.62, respectively. The VIFs were less than 10, indicating the absence of multicollinearity. The model met the requirements. The regression analysis results showed a significant negative correlation between AMS and BFS-P scores (*β* = -27.54, *p* = 0.03) and a significant positive correlation between AMS and BFS-N scores (*β* = 21.97, *p* = 0.03). This means that after developing AMS, positive emotions decreased by 27.54, while negative emotions increased by 21.97 (Table 2).

**Table 2:** Coefficient test for regression models of positive emotions and negative emotions.

Model		Unstandardized coefficients		Standardized coefficients	*t*	*P*	*R*^2^	Adjusted R square	*F*	Collinearity diagnosis	
		*β*	Std. Error	Beta						VIF	Tolerance
BFS-P	Constant	62.21	5.81	–	10.71	0.00**	0.38	0.31	5.31**	–	–
	AMS	−27.54	12.08	−0.93	−2.28	0.03*				6.93	0.14
	LLS score	1.46	1.81	0.34	0.81	0.43				7.60	0.13
	Altitude	0.34	3.10	0.02	0.11	0.92				1.37	0.73
BFS-N	Constant	27.27	4.67	–	5.84	0.00**	0.65	0.62	16.41**	–	–
	AMS	21.97	9.71	0.69	2.26	0.03*				6.93	0.14
	LLS score	0.51	1.46	0.12	0.37	0.71				7.60	0.13
	Altitude	0.45	2.49	0.02	0.18	0.86				1.37	0.73

*p < 0.05.

**p < 0.01.

During the 3-day mountaineering activity, 5 individuals developed AMS on both Day 2 and Day 3, and they were the same individuals. According to the LLS scores, AMS was more severe on Day 3, with 3 individuals showing a significant increase in LLS scores. Symptoms gradually improved or disappeared after the patients descended to lower altitudes. Using the Wilcoxon rank-sum test, the emotions of AMS+ and AMS− individuals at different altitudes during the 3-day period were examined. The results showed that on Day 2, at an altitude of 4300 m, there was a highly significant difference in the depressive subscale score on the BFS between the AMS+ and AMS− groups (*p* < 0.01). Significant differences (*p* < 0.05) were also observed in the subscales of anger, agitation, and inactivity. On Day 3, upon returning to an altitude of 3140 m, significant differences (*p* < 0.05) were found in the scores for the subscales of happiness, calmness, anger, agitation, and depression (Table 3).

**Table 3:** Changes in emotions for AMS+ and AMS− at different altitudes.

Date	Number	Ground	LLS	Activity	Happiness	Thoughtfulness	Calmness	Anger	Agitation	Depressive	Inactivity
Day 1	0	AMS+	0	0	0	0	0	0	0	0	0
	10	AMS−	0 (0,1)	19.5 (17.25,21.5)	19.5 (16.75,21)	10.5 (9.75,13.5)	18 (15.75,19.5)	6 (5,7)	8.5 (6.75,11.5)	6 (5,7.25)	6 (5,8.25)
		*P*	*–*	*–*	*–*	*–*	*–*	*–*	*–*	*–*	*–*
Day 2	5	AMS+	6 (5,7.5)	9 (5.5,11)	11 (7.5,12)	12 (10,15)	10 (6.5,14)	12 (8.5,13.5)	12 (11,17)	15 (9,15.5)	16 (11.5,17.5)
	5	AMS−	1 (0,1.5)	10 (9,17.5)	11 (8,16.5)	13 (9,14.5)	14 (10.5,16)	8 (5,9)	6 (5,10.5)	7 (5,8)	8 (5,11.5)
		*p*	0.008**	0.243	0.598	0.915	0.207	0.044*	0.046*	0.008**	0.036*
Day 3	5	AMS+	9 (5.5,10)	14 (5,16.5)	10 (8,15.5)	12 (9.5,17)	14 (12.5,18)	12 (7.5,15.5)	11 (8.5,15.5)	16 (10.5,18)	18 (14.5,21.5)
	5	AMS−	1 (1,1)	20 (11.5,20.5)	20 (18,22)	12 (10.5,17.5)	21 (16.5,22)	5 (5,7.5)	6 (5.5,8.5)	5 (5,7.5)	6 (5,15.5)
		*P*	0.005**	0.071	0.016*	0.673	0.046*	0.024*	0.044*	0.013*	0.074

* p < 0.05.

** p < 0.01.

**Figure 2:**
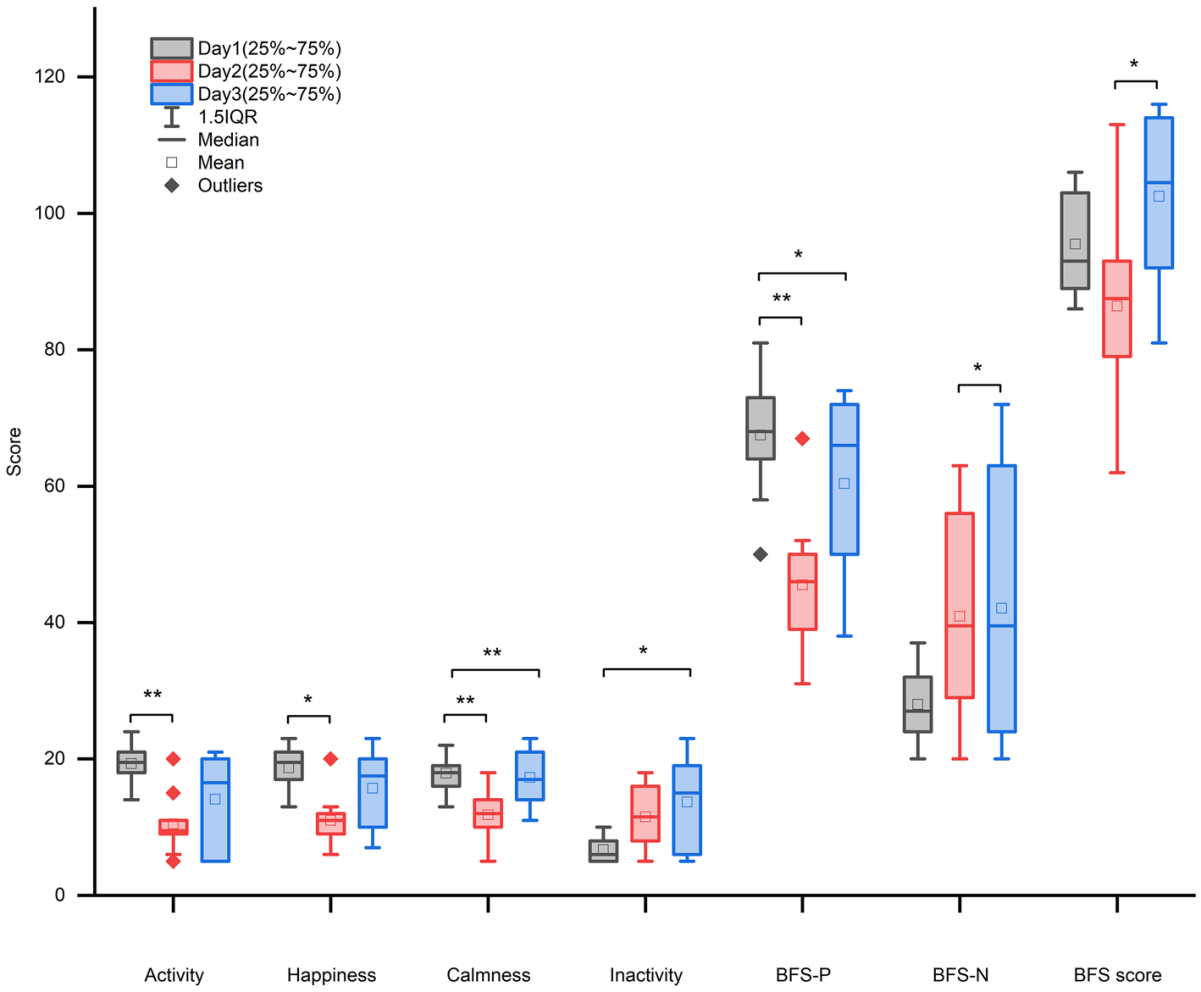
Nemenyi test.

Table 4 presents the results of the Friedman test comparing the emotions measured by the BFS subscales at different altitudes. The Nemenyi test was used to further compare the differences in BFS subscales among different altitudes and different stages of mountaineering activity. The results revealed significant differences in activity, happiness, calmness, inactivity, BFS-P, BFS-N, and BFS score among the different altitudes (*p* < 0.05). The pairwise comparisons revealed significant differences in activity, happiness, calmness, BFS-P, and BFS score between Day 1 and Day 2 (*p* < 0.05); significant differences in calmness and BFS-P between Day 1 and Day 3 (*p* < 0.05); and significant differences in calmness and BFS-N and BFS scores between Day 2 and Day 3 (*p* < 0.05) (Fig. 2).

**Table 4:** Differences in BFS subscales at different altitudes.

BFS subscale	Date	Median (P25, P75)	χ^2^	*p*
Activity	1	19.5 (18.25,20.75)	11.842	0.003**
	2	9.5 (9,11)		
	3	16.5 (7.25,19.5)		
Happiness	1	19.5 (17,20.75)	9.556	0.008**
	2	11 (9.25,12)		
	3	17.5 (10.75,19.75)		
Thoughtfulness	1	10.5 (10,11.75)	2.057	0.358
	2	12.5 (9.5,13)		
	3	12 (10.25,16)		
Calmness	1	18 (16.5,19)	12.789	0.002**
	2	12 (10,14)		
	3	17 (14.25,20.75)		
Anger	1	6 (5,6.75)	5.471	0.065
	2	8.5 (8,11.5)		
	3	7.5 (5,11.5)		
Agitation	1	8.5 (7,10.75)	0.839	0.657
	2	11 (6.5,12.75)		
	3	8.5 (6,11)		
Depressive	1	6 (5,7)	5.871	0.053
	2	8.5 (7.25,13.5)		
	3	8.5 (5,15.5)		
Inactivity	1	6 (5.25,7.75)	9.75	0.008**
	2	11.5 (8,15.75)		
	3	15 (7.25,18.75)		
BFS-P	1	68 (64.5,72.75)	12.053	0.002**
	2	46 (39.75,50)		
	3	66 (50.25,71.5)		
BFS-N	1	27 (24.5,31.75)	7.371	0.025*
	2	39.5 (30.75,53)		
	3	39.5 (26.75,58.25)		
BFS score	1	93 (89.5,102.75)	6.821	0.033*
	2	87.5 (79.5,92)		
	3	104.5 (92.5,113.75)		

*p = 0.05.

**p = 0.01.

## Discussion

This study aimed to investigate the emotional states of ten amateur mountaineers during their ascent to high altitudes and explore the factors influencing their emotions in a real high-altitude environment. The findings indicated a deterioration of emotions with increasing altitude, followed by improvement during descent. Significant differences in negative emotions were observed between AMS+ and AMS− individuals. Furthermore, AMS emerged as a predictive factor for emotional states.

**Figure 3:**
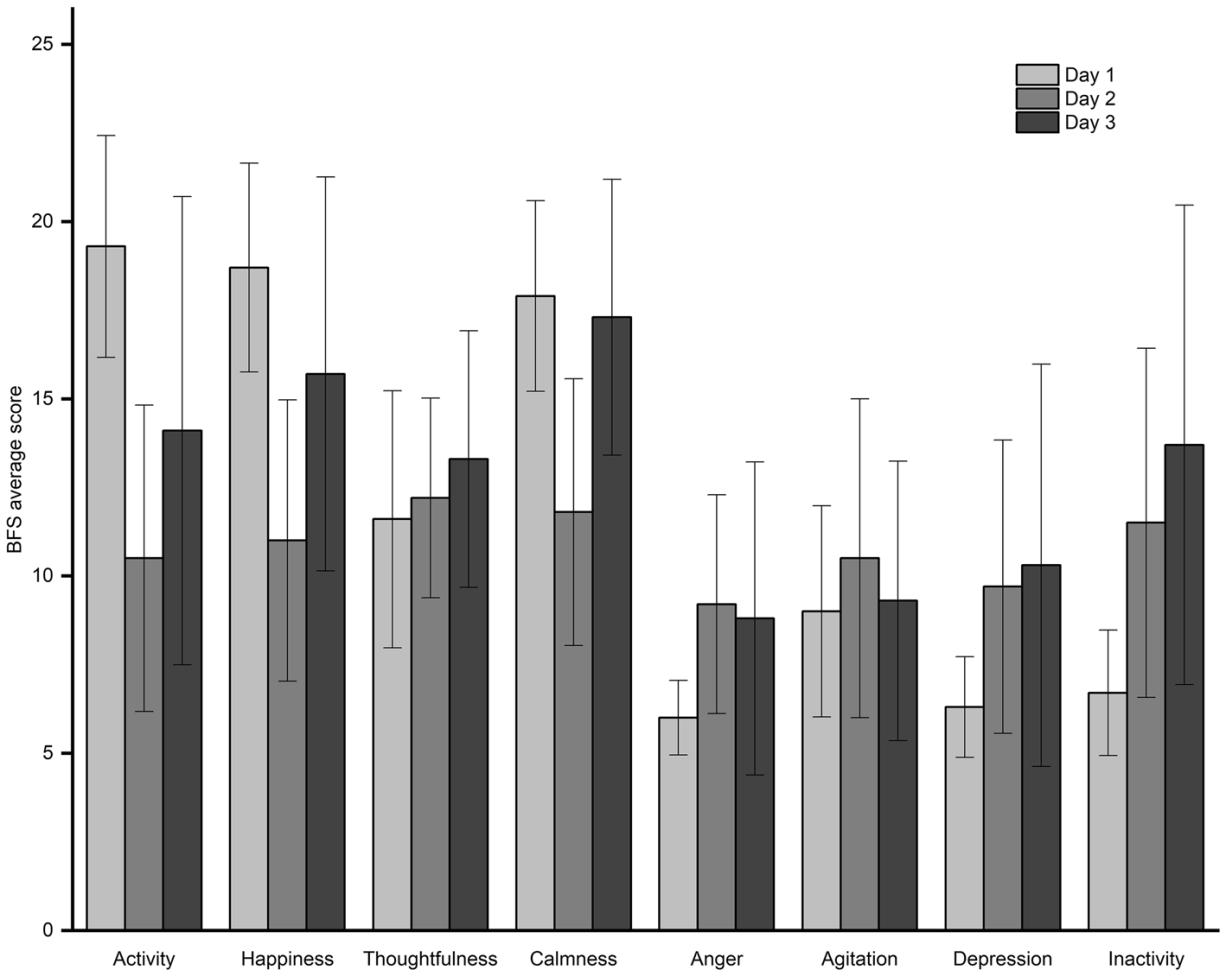
Changes in the BFS subscale on the 3rd day.

The findings of this study contribute to our understanding of the emotional experiences of amateur mountaineers in high-altitude environments. The results indicate a pivotal role of altitude in shaping emotions, with higher elevations correlating with emotional deterioration, consistent with prior research emphasizing the impact of altitude on emotional well-being^14,15^. The emotions of the ten amateur mountaineers exhibited peaks and valleys during the mountaineering process^26^. As they ascended from 3140 to 4300 m, there was a significant decrease in the scores on the subscales of activity, happiness, and calmness within the positive emotions, and a significant increase in the score on the subscale of passivity within the negative emotions. Although the remaining BFS subscales did not significantly change, there was an overall upward trend in negative emotions (Fig. 3). The negative emotions of the AMS+ individuals on Day 2 and Day 3 differed significantly from those of the AMS− individuals. Given the wide range of emotional experiences and individual differences^27^, this study provides only a general discussion.

### The impact of acute mountain sickness on emotions

When individuals enter high-altitude environments, they are immediately confronted with stressors that affect their physical and psychological well-being. Hypoxia, a distinct feature of HA regions, has been identified in the literature as a significant contributor to emotional disturbances^11,28^. Furthermore, hypoxia can impact neurotransmitter synthesis, which is considered a fundamental cause of emotional disturbances^29^ and can also lead to Acute Mountain Sickness (AMS). The probability of experiencing AMS increases when mountaineers reach altitudes of 2500 m or higher due to rapid ascent and physical exertion^30^. Hypoxia combined with prolonged and intense mountaineering exacerbates fluid loss in the body, leading to increased fatigue and a greater risk of developing AMS^31^. Emotions also have a reciprocal relationship with AMS, as they significantly influence individual symptom reporting^32^.

This study revealed that individuals with AMS+ exhibit more negative emotions than those with AMS−. The main manifestations of negative emotions include anger, excitement, depression, and fatigue. Physical activity in hypoxic environments leads to an increase in negative affect^33^. Individuals with a history of mental health conditions are more likely to develop AMS^34^, and heightened AMS severity is correlated with negative affect^35^. Our findings align with Bian, et al.^36^ report, demonstrating a positive correlation between AMS scores and negative affect, indicating fewer positive emotions and more negative emotions in AMS+ patients. After a 3-month observation of mountaineers attempting to summit Mount Everest, significant decreases in trait anxiety, feelings of tension, and fatigue were observed after the summit was reached, while positive emotions showed an increasing trend^10^. However, additional studies have reported negative emotions as the predominant emotional state during mountaineering, which is highly correlated with an individual's mountaineering experience^37^. In our study involving amateur mountaineers, we observed an exacerbation of AMS severity after a 15-h stay at 4300 m. Although there was an increase in positive emotions after participants reached the summit, the magnitude of the increase was not significant. In contrast, AMS+ individuals experienced peak levels of anger, depression, and inactivity, in clear contrast to professional mountaineers.

In conclusion, the observed significant differences in negative emotions between AMS+ and AMS− individuals underscore the pivotal role of AMS status in influencing emotional states during mountaineering activities. Individuals with AMS experienced higher levels of negative emotions, such as anger, agitation, and depression. This finding emphasizes the need for adequate measures to prevent and manage AMS among mountaineers, as it not only affects physical health but also has a profound impact on emotional well-being. Moreover, the predictive role of AMS for emotions highlights its potential as an early indicator of emotional changes in high-altitude environments. These findings suggest that monitoring and addressing AMS symptoms could help mitigate the negative emotional impact experienced by mountaineers.

### Effect of altitude on emotion

There are three main perspectives on the emotional changes that occur at specific altitudes: above 3000 m^16^, above 3500 m^17^, and above 4000 m^18,19^. Our study aligns with the conclusion of Shukitt and Banderet^18^, who suggested that emotions deteriorate at altitudes above 4000 m. Given the short duration and rapid ascent in a single day in our study, positive emotions remained dominant overall. This phenomenon may be attributed to the fact that all participants were first-time mountaineers. However, it cannot be ruled out that the increased fatigue experienced by mountaineers due to the prolonged and intense nature of the climb suppresses negative emotions^38^. In the case of amateur mountaineers, we observed that upon entering HA regions, positive emotions predominated. However, with time and increasing altitude, emotions gradually deteriorate, consistent with the emotional trend observed by Banderet and Shukitt-Hale^39^. Notably, prolonged stays in high-altitude regions can lead to negative effects such as hostile emotions and impaired thinking^18^.

This study revealed that staying at high altitudes and ascending to elevation can lead to emotional disorders. The longer the duration of stay at high altitudes is, the more the emotional state improves^10^. However, these methods are limited by their ability to stay at the same altitude, and increased elevation can still cause deterioration in mood. Various factors affect mood during mountaineering. Ruffini and Cera^31^ reported that constant physical exertion during mountaineering can lead to a decrease in positive mood, while excessive physical exertion may trigger the production of androgenic anabolic steroids, leading to significant increases in irritability and aggression, which in turn can trigger negative moods^40^. Additionally, the wilderness, characterized by sparsely populated and socially isolated high altitudes, leads to monotony, which may adversely affect human physiology and lead to mood changes, especially AMS. Monotony may adversely affect human physiology and lead to altered mood, especially AMS, while lack of guidance, language barriers, culture shock, and unknown risks during mountaineering may alter mood.

Although the participants in this study did not stay at high altitudes for an extended period, the positive emotions of the mountaineers improved on the day they finished mountaineering and descended in altitude. This improvement was primarily characterized by an increase in positive emotions, though not necessarily a return to the initial level from the first day. Notably, on the day of descent to 3140 m on the third day after the summit was reached, there was no significant decrease in negative emotions; instead, symptoms of depression and lack of vitality increased. In summary, ascending elevation causes emotional changes in mountaineers, and their emotional state continues to be influenced by negative emotions in the short term after completing the climb.

### Limitations and prospects

It is worth noting that this study has several limitations. The small sample size and focus on amateur mountaineers restrict the generalizability of the research findings. However, further research is needed to confirm and extend these results by studying larger and more diverse samples. Additionally, the study did not examine other potential factors that may influence emotions during mountaineering activities, such as individual differences or environmental conditions. Future research should aim to replicate these findings with larger samples and consider additional factors to gain a more comprehensive understanding of emotional dynamics in high-altitude mountaineering.

## Conclusion

This study provides valuable insights into the emotional experiences of amateur mountaineers in high-altitude environments. The research findings indicate that altitude plays a significant role in shaping emotions, with AMS status being a key factor. Based on this study, we propose the following recommendations: prior to mountaineering, enhance adaptability to high-altitude environments and improve psychological resilience through training. Maintaining stable emotions can promote better performance among mountaineers^13^ and increase mountaineering safety. During the climb, screening and monitoring of emotional changes should be strengthened, and interventions and adjustments should be applied promptly if deterioration is detected. After climbing, individuals should actively intervene to alleviate the enduring impact of negative emotions on mountaineers.

### Supplementary Information

Supplementary Information.

## Data Availability

All data generated or analysed during this study are included in this published article (and its Supplementary Information files).
